# Obesity hypoventilation syndrome, literature review

**DOI:** 10.1093/sleepadvances/zpae033

**Published:** 2024-06-08

**Authors:** Bertha Nachelly Orozco González, Nidia Rodriguez Plascencia, Julio Augusto Palma Zapata, Alondra Esthefanía Llamas Domínguez, Jesús Sacramento Rodríguez González, Juan Manuel Diaz, Miguel Ponce Muñoz, Silvia Denise Ponce-Campos

**Affiliations:** Pneumology Service, Hospital of Specialties at the National Medical Center of the West (IMSS), Guadalajara, México; Pneumology Service, Hospital of Specialties at the National Medical Center of the West (IMSS), Guadalajara, México; Medical Didactic Unit, Autonomous University of Aguascalientes, Aguascalientes, México; Medical Didactic Unit, Autonomous University of Aguascalientes, Aguascalientes, México; Department of Medicine, Autonomous University of Aguascalientes, Aguascalientes, México; Department of Microbiology and Immunology, University of Western Ontario, London, ON, Canada; Department of Medicine, Autonomous University of Aguascalientes, Aguascalientes, México; Pneumology Service, Institute of Security and Social Services for State Workers, Aguascalientes, México

**Keywords:** hypoxia, obesity, sleep disordered breathing, chronic fatigue, sleepiness, OSA

## Abstract

Obesity is a global health concern that has been increasing over the years, and it is associated with several pathophysiological changes affecting the respiratory system, including alveolar hypoventilation. Obesity hypoventilation syndrome (OHS) is one of the six subtypes of sleep-hypoventilation disorders. It is defined as the presence of obesity, chronic alveolar hypoventilation leading to daytime hypercapnia and hypoxia, and sleep-disordered breathing. The existence of a sleep disorder is one of the characteristics that patients with OHS present. Among them, 90% of patients have obstructive sleep apnea (OSA), and the remaining 10% of patients with OHS have non-obstructive sleep hypoventilation without OSA or with mild OSA. This review aims to provide a comprehensive understanding of the epidemiological and pathophysiological impact of OHS and to highlight its clinical features, prognosis, and severity, as well as the available treatment options.

**Statement of Significance**

This article aims to highlight the criticality of timely diagnosis of obesity hypoventilation syndrome in the clinical setting and the significance of synthesizing available information to achieve optimal treatment outcomes. It is worth noting that obesity hypoventilation syndrome is often neglected, particularly in preoperative assessment. Therefore, a thorough knowledge is essential. Moreover, treatment of this disease has been controversial over the years. In this regard, various treatment options are available, and the presented data in this work provides guidance through specific strategies. Thus, this review emphasizes all the points with a practical approach while providing a comprehensive overview of the topic.

Obesity is a global health concern that has been on the rise over the years and is associated with different conditions that can adversely impact the morbidity and mortality of populations. According to the World Health Organization (WHO), in 2016, more than 1.9 billion adults aged 18 and above were overweight, and out of these, 650 million were obese. In the United States, the prevalence of overweight and obesity has tripled in the last 50 years, with a reported prevalence of 62.5% in adults over the age of 18 in 2016, of which 28.6% were obese, making it the highest regional prevalence in the world. Additionally, the United States and Mexico have the highest prevalence of obesity, with 42.4% and 36.9%, respectively [1].

According to the “Encuesta Nacional de Salud y Nutrición” (ENSANUT) conducted in Mexico in 2022, there is a considerable prevalence of overweight and obesity in the adult population. The survey reported a prevalence of overweight of 38.3%, with a higher rate in men (41.2%) compared to women (35.8%). Similarly, 36.9% of adults were obese, with a higher rate in women (41%) compared to men (32.3%) [2].

Obesity is associated with several pathophysiological changes that affect the respiratory system, including alveolar hypoventilation. Alveolar hypoventilation is characterized by increased alveolar partial pressure of carbon dioxide (PaCO2), leading to hypercapnia and acid-base disorders. The body compensates for these changes chronically by varying bicarbonate levels. Reduction in minute ventilation is a common feature of all chronic hypoventilation disorders and leads to daytime hypercapnia. Minute ventilation is regulated according to the prevailing carbon dioxide (CO2) levels, and decreases (hypoventilation) in minute ventilation leads to the accumulation of carbon dioxide. There are two different issues that can lead to obesity hypoventilation syndrome (OHS). Firstly, it can occur due to failure of respiratory regulation caused by insufficient respiratory drive in the brain stem. Secondly, it can happen due to the execution of impulses resulting from impaired transmission of the breathing impulses in the spinal cord and peripheral nerve, and morphological or functional abnormalities of the musculoskeletal system of the thorax. OHS is a combination of these two pathomechanisms [3].

The International Classification of Sleep Disorders includes six subtypes of hypoventilation disorders in the chapter on sleep-related breathing disorders: OHS, congenital alveolar hypoventilation due to late hypothalamic dysfunction, idiopathic alveolar hypoventilation, hypoventilation during sleep associated with substances or medications, and hypoventilation during sleep due to other diseases [4].

This review is focused primarily on OHS, with the objective of generating an early identification and a thorough understanding of the issues that will help identify hypoventilation, which will allow proper management of this disease, and concluding with key findings from this review.

Conducting a bibliographic review of OHS is of utmost significance, given its tendency to be underdiagnosed. It is imperative to note that early identification of OHS is crucial to mitigate the severity of associated comorbidities and to prevent mortality. Therefore, raising awareness about the importance of early identification of OHS is essential in clinical settings.

## History

In 1837, Charles Dickens referred to a character named Joe in one of his works, describing him as an individual who was overweight, had a rosy complexion, snored, struggled to breathe, and felt constantly drowsy [5]. Subsequently, in 1889, the first literature report was published linking excess weight to excessive sleepiness. Lavie P. was the first to coin the term “Pickwick” about a sleep disorder and the first to use the term Pickwick associated with sleeping sickness [6]. In 1909, the then President of the United States, Howard Taft, compared himself to the character Joe from Charles Dickens’ literary due to his overweight condition. Taft noted that his symptoms of drowsiness and fatigue had improved significantly, in addition to observing changes in his skin color after losing about 40 kg (88.18 lb) [7]. However, the first well-documented case of a patient with OHS in the medical literature was described by Auchincloss and colleagues in 1955; they described the clinical and physiological aspects of a patient with obesity who presented erythrocytosis and hypoventilation [8]. After a year, the term “Pickwick Syndrome” gained popularity thanks to Burwell. He recollected a patient who bore a resemblance to the fat boy Joe. The patient exhibited drowsiness, hypoventilation, and signs of cor pulmonale. After losing 18 kg, the patient’s PaCO2 and blood pressure levels were normalized [9].

Research on OHS is ongoing, and several aspects of this syndrome still require further investigation. However, we can identify two critical events in the history of OHS that have significantly impacted its treatment and understanding. The first event was Sullivan’s invention of positive pressure equipment in 1983, which opened new avenues for treatment beyond tracheostomy. The second event was the discovery of leptin in 1994 by Jeffrey Freidman. This groundbreaking discovery led to an understanding of many of the pathophysiological mechanisms underlying OHS [10, 11].

## Definition

**Figure 1.**
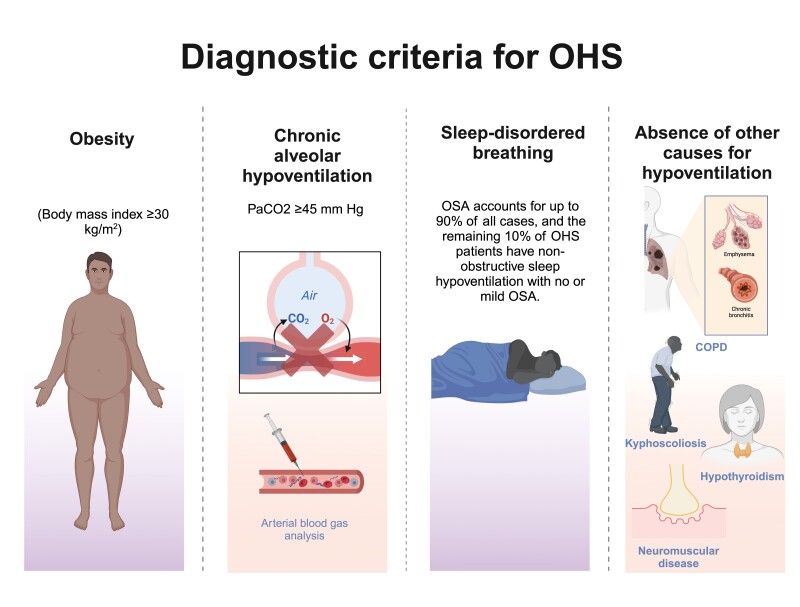
Diagnosis of obesity hypoventilation syndrome. The diagnosis is made by the combination of obesity, sleep-disordered breathing, and daytime hypercapnia in the absence of a neuromuscular, mechanical, or metabolic cause of hypoventilation (some of these conditions include chronic obstructive pulmonary disease, restrictive lung disease, kyphoscoliosis, hypothyroidism, neuromuscular diseases, and central hypoventilation). COPD, chronic obstructive pulmonary disease; OHS, obesity hypoventilation syndrome; OSA, obstructive sleep apnea; PaCO2, arterial carbon dioxide tension; PaO2, partial pressure of oxygen.

OHS is defined by the presence of obesity (body mass index [BMI] ≥ 30 kg/m2), chronic alveolar hypoventilation leading to daytime hypercapnia (PaCO2 ≥ 45 mmHg) as well as sleep-disordered breathing (SDB), after excluding other causes for hypoventilation (such as neuromuscular, metabolic, lung, or chest wall diseases; **Figure 1**) [12, 13]

The existence of a sleep disorder is one of the characteristics that patients with OHS present. Within these, obstructive sleep apnea (OSA) is the most prevalent respiratory disorder occurring during sleep and accounts for up to 90% of all cases. The remaining 10% of OHS patients have non-obstructive sleep hypoventilation with no or mild OSA [12].

OSA is a sleep disorder characterized by the repeated collapse of the upper airways, leading to partial or complete airway blockage. This can result in decreased oxygen levels and interrupted sleep, causing fatigue, sleepiness, and other symptoms affecting quality of life. The diagnosis of OSA is determined by the presence of related symptoms, such as sleepiness, habitual snoring, gasping, breath-holding, or choking during sleep, as well as the identification of more than five predominantly obstructive respiratory events/hour (obstructive and mixed apneas, hypopneas, or respiratory effort related arousals) measured by polysomnography or out of center sleep testing (apnea–hypopnea index (AHI) of ≥ 5 events/h). Alternatively, a diagnostic criterion of an obstructive respiratory event rate of 15 events/h can be used, even without symptoms or associated comorbidities. An AHI ≥ 30 events/h is primarily classified as severe [14, 15].

According to the American Academy of Sleep Medicine, this sleep-related hypoventilation is defined as the presence of PaCO2 of greater than 55 mmHg for over 10 minutes or an increase in PaCO2 by greater than 10 mmHg during sleep, in comparison to a PaCO2 more significant than 50 mmHg for more than 10 minutes in the awake supine value [16, 17].

## Epidemiology

The increasing prevalence of obesity has led to a corresponding rise in the occurrence of OHS, not only among adults but also among children and adolescents. Epidemiological studies conducted on different populations have demonstrated that the prevalence of OHS in patients with obstructive sleep apnea-hypopnea syndrome (OSAHS) and obesity ranges from 10% to 20% [18]. This percentage can escalate to 27% in patients with a BMI greater than 40 kg/m2 and even as high as 50% in those with a BMI greater than 50 kg/m2 [19]. Thus, the prevalence of OHS is directly related to the prevalence of obesity within each population. For example, the prevalence of OHS in patients with OSA in Japan is only 9% [20]. In contrast, it reaches 20% in the United States, which is unsurprising given that the United States has the highest obesity rate globally [21]. In fact, one-third of the US population has a BMI above 30 kg/m2 [22, 23].

However, the prevalence of OSAHS in the Mexican population stands at 3.3%, with men being more affected than women at a rate of 4.4% and 2.2%, respectively. However, the prevalence of OHS is presently unknown [24]. The association between gender is not clearly defined; some research findings suggest that it is more prevalent in men [25], while others claim it is more common in women [26]. Additionally, there is not a higher incidence in any race or ethnicity. However, OHS occurs at a lower BMI range in the Asian population [12, 27].

Regrettably, most cases of OHS are diagnosed belatedly, typically during the fifth or sixth decade of the patient’s life, when hospitalization is required because of some complication or another [28]. This presents a significant challenge, and the multidisciplinary medical staff must remain vigilant, especially given the continuing rise in the prevalence of obesity [23].

## Pathophysiology

OHS is a medical condition that results from changes in the respiratory system due to excess adipose tissue. Pathophysiological mechanisms responsible for this syndrome have recently been proposed. The increase in chest wall thickness reduces lung volume, leading to a decrease in the functional residual capacity (FRC) [29]. This can impede the muscular function of the diaphragm, decreasing pulmonary adherence and increasing the resistance of the lower airway. The characteristic breathing pattern that arises because of alveoli closing prematurely during expiration includes low tidal volume and increased respiratory rate. This heightened respiratory rate further contributes to increased dead space ventilation. Additionally, the decreased ventilation of the lower lobes and the FRC reduction may lead to changes in ventilation-perfusion (V/Q), ultimately resulting in hypoxemia [16].

Another explanation is alterations in the respiratory drive; obese patients tend to increase their respiratory capacity to maintain normal CO2 levels in their bodies. However, alterations in their respiratory drive can lead to hypoventilation, especially during rapid eye movement sleep. During this stage of sleep, there is a general relaxation of the muscles, and the diaphragm and central impulses control ventilation. If this pattern continues, it can lead to a secondary depression of the respiratory centers, resulting in hypercapnia during the day. This could explain the high prevalence of central hypoventilation in the OHS [3]. Eucapnic patients can normalize the PaCO2 levels via compensatory augmentation of alveolar ventilation, which increases CO2 clearance; but in OHS patients, the compensatory mechanism is disrupted, causing the retention of CO2. In response to CO2 accumulated beyond the ventilatory capacity to be cleared, the renal system decreases bicarbonate clearance to compensate for the hypercapnic pH drop. This built-up in bicarbonate eventually blunts the ventilatory response to CO2, thus causing the development of nocturnal hypoventilation. This chronic accumulation of CO2 leads to chronic hypercapnia and compensated respiratory acidosis [30].

Some studies have demonstrated the role of leptin as a respiratory stimulant, and how leptin resistance related to obesity can contribute to a decline in respiratory control. Furthermore, insulin-like growth factor has been identified as a possible factor in developing this condition [12].

**Figure 2.**
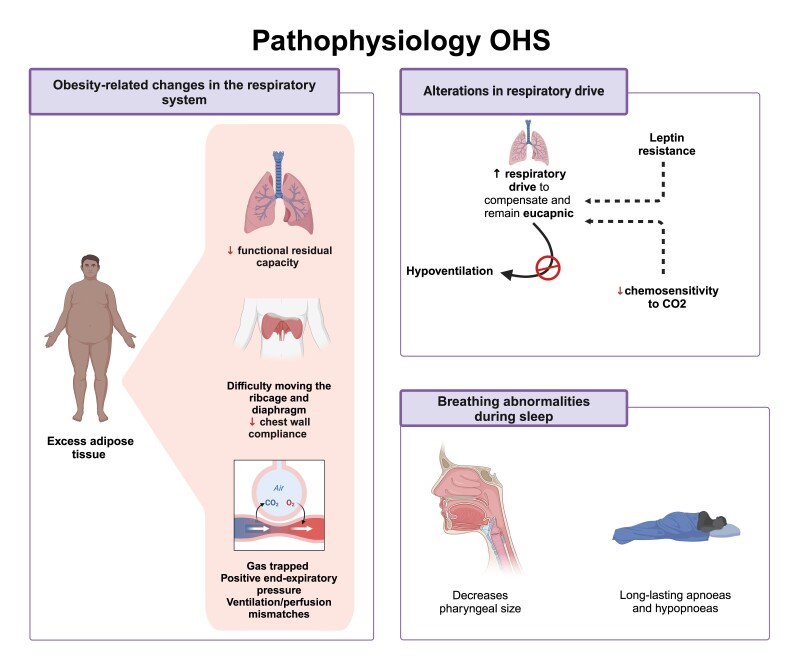
Main pathophysiological mechanisms of OHS. OHS is a medical condition caused by excess body fat, which changes the respiratory system, reducing lung volume and causing air trapping. Alterations in respiratory drive and leptin resistance are factors that contribute to the development of this condition. Patients with OHS experience obstructive apneas and hypopneas during supine sleep.

Another explanation is the alteration of respiratory functions during sleep, respiratory alterations can occur due to the physiological changes in obesity amplified during supine sleep. The excessive fat surrounding the upper airway and the reduction in lung volume can cause the pharynx to collapse [31]. Patients with OHS have been observed to experience obstructive apneas and hypopneas of longer duration compared to patients with OSA alone, indicating a poorly compensated ventilatory response. (**Figure 2**) [32]. Patients with obesity may also present with diastolic ventricular dysfunction, which increases their risk of developing post-capillary pulmonary hypertension [31].

It is noteworthy that a significant proportion of these patients display hypoxemia, which has been linked to hypercapnia during the day. Such sustained hypoxia may delay sleep activation in response, as observed in clinical studies [33].

## Prognosis (Morbidity and Mortality)

As is well established, patients diagnosed with OSAHS are at an increased risk of developing chronic degenerative diseases, such as metabolic, cerebrovascular, and coronary diseases. This can lead to higher mortality rates [34–40].

In contrast, patients diagnosed with OHS, despite having the same degree of obesity as those with OSAHS, usually experience more significant morbidity and mortality rates if left undiagnosed and untreated promptly. Additionally, the quality of life of such patients is often worse, both compared to obese patients without hypercapnia and those with hypercapnia due to other causes, mainly due to excessive sleepiness [41, 42].

### Morbidity

Patients with obesity and concomitant hypercapnia have a higher risk of morbidity compared to those without hypercapnia. Patients with OHS exhibit a higher frequency of medical consultations, emergency services usage, more extended hospital stays, intensive care unit (ICU) admissions, invasive mechanical ventilation requirements, and perioperative complications [23].

In comparison to obese individuals without hypoventilation, patients with OHS are at a higher risk of heart failure (OR 9, 95% CI: 2.3 to 35), angina pectoris (OR 9, 95% CI: 1.4 to 57.1), and cor pulmonale (OR 9, 95% CI: 1.4 to 57.1) [26].

OHS patients also exhibit a greater prevalence of pulmonary hypertension, reportedly up to 88%, compared to those with only OSAHS, which is 15%. Most OHS patients belong to group III, associated with chronic hypoxemia, while a minority is within group II, associated with left heart failure or obese cardiomyopathy [43]. Moreover, this patient group has a higher prevalence of systemic arterial hypertension (55%–88%), diabetes mellitus, and osteoarthritis [44].

Appropriate diagnosis and treatment of OHS can significantly reduce hospital stay from 7.9 to 2.5 days per year [26].

### Mortality

The primary causes of mortality in OHS patients include hypercapnic respiratory failure, exacerbated cor pulmonale, or pulmonary thromboembolism [45]. Studies indicate that patients with OHS who decline continuous positive airway pressure (PAP; CPAP) management have an alarmingly high mortality rate of up to 46% within 50 months of follow-up [46]. Furthermore, hospitalized patients who do not receive treatment have a mortality rate of 23% at 18 months, compared to only 6% in obese patients without hypercapnia. However, when treated with noninvasive ventilation (NIV), the mortality rate at 18 months is reduced to only 3%, with rates of 8% and 30% at 2 and 5 years, respectively [23]. Early identification and treatment can prevent hospital admissions, ICU stays, the use of mechanical ventilation, and death [47].

## Diagnosis and clinical manifestations

The diagnosis of this condition typically emerges in individuals between 40 and 60 years of age. There exists a delay in diagnosis, as approximately 75% of patients are often misdiagnosed with chronic obstructive pulmonary disease [32].

The clinical manifestations typically exhibit symptoms and signs like those of patients with OSAHS. These include habitual snoring, witnessed apneas, associated asphyxia awakenings, excessive daytime sleepiness, mood disorders, and morning headaches [47, 48].

However, in contrast to patients with eucapnic OSAHS, most OHS patients often present with signs of cor pulmonale, such as dyspnea, facial redness, cyanosis, jugular engorgement, reinforcement of the second heart sound (challenging to auscultate due to obesity), discrete edema of the lower extremities, evidence of venous insufficiency of the lower extremities, erythrocytosis, hypoxemia associated with hypercapnia, and even a higher degree of obesity (generally BMI > 40 kg/m2), neck circumference and drowsiness [47, 48].

Patients with OHS can present to healthcare providers in one of the two ways. The first presentation involves a patient in an exacerbated state, presenting with respiratory acidosis and signs of right heart failure. This type of patient requires management in the ICU. They often require NIV and, in some cases, may require oral intubation and invasive ventilation. In severe cases, a tracheostomy may be necessary. The second presentation typically involves a stable patient who seeks medical attention from a pulmonologist or sleep specialist. Healthcare providers need to recognize both presentations of OHS to diagnose and treat patients properly [23, 25, 28].

As highlighted previously, the diagnosis of OHS is often delayed. This delay is attributed to medical consultations typically employing a pulse oximeter to assess oxygen saturation (SpO2) alone, neglecting the potential presence of hypercapnia at the disease’s onset, even in the absence of hypoxemia. Consequently, patients may receive supplemental oxygen without addressing the underlying hypoventilation. Therefore, it is recommended that an arterial blood gas analysis be performed to evaluate PaCO2 whenever an obese patient presents with unexplained hypoxemia and cor pulmonale data [19]. This evaluation will help identify hypoventilation, enabling appropriate care. For patients with OSA, it is recommended that medical professionals measure arterial blood gas to confirm hypercapnia if their serum bicarbonate level is 27 mmol/L or higher and their nadir SpO2 is less than 80%. Measuring serum bicarbonate levels can be a less invasive initial screening method [49]. According to some experts, a serum bicarbonate threshold of 27 mEq/L has a 92% sensitivity and 50% specificity in detecting hypercapnia among patients with OHS [21].

A recent cohort study was conducted to determine the common characteristics of patients diagnosed with OHS. The study revealed that most patients with this condition were male individuals between the ages of 42 and 61, with a BMI ranging from 35 to 56 and a neck circumference between 45 and 47 cm. The arterial blood gas analysis reported that their pH was 7.34 to 7.40, while their PCO2 and PO2 levels were between 47 and 61 mmHg and 46–74 mmHg, respectively. The study also found that the bicarbonate level was 31–33 mEq/L, while the AHI ranged from 20 to 100. The saturation time below 90 (T90) was between 46% and 56%, while the forced vital capacity ranged from 57% to 102%. Additionally, the forced expiratory volume in the first second (FEV1) was found to be between 53% and 92%, while the FEV1/forced vital capacity ratio ranged from 0.74 to 0.88. Lastly, the Epworth Sleepiness Scale score of these patients was found to be between 12 and 16 [50]. These findings provide valuable insights into the characteristics of patients, which may help clinicians better understand and manage this condition.

The definitive diagnostic test for OHS involves measuring PCO2 levels through arterial blood gas analysis (without supplemental oxygen). However, there may be occasions where this test could be impractical, and a reliable alternative is to measure CO2 levels through transcutaneous or end-expiratory monitoring. Such a method is available during polysomnography or respiratory polygraphy, and it provides valuable insights into the presence of respiratory sleep disorders, including OSAHS. Furthermore, this method assists in titrating subsequent treatments for this condition, which will be discussed in the following section [32].

## Treatment

**Figure 3.**
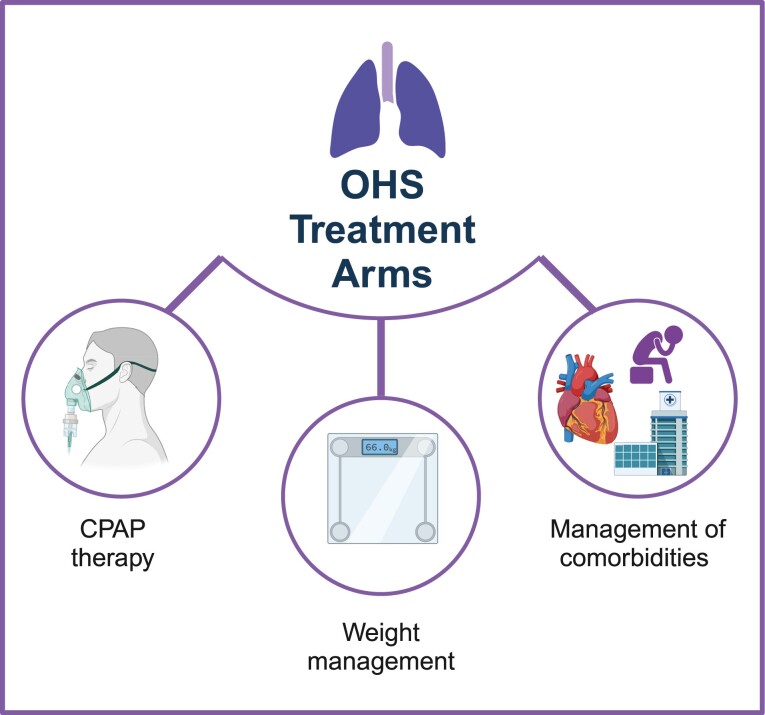
The mainstays of OHS treatment. The treatment approach involves correcting sleep disordered breathing, reducing weight, and managing comorbidities.

**Figure 4.**
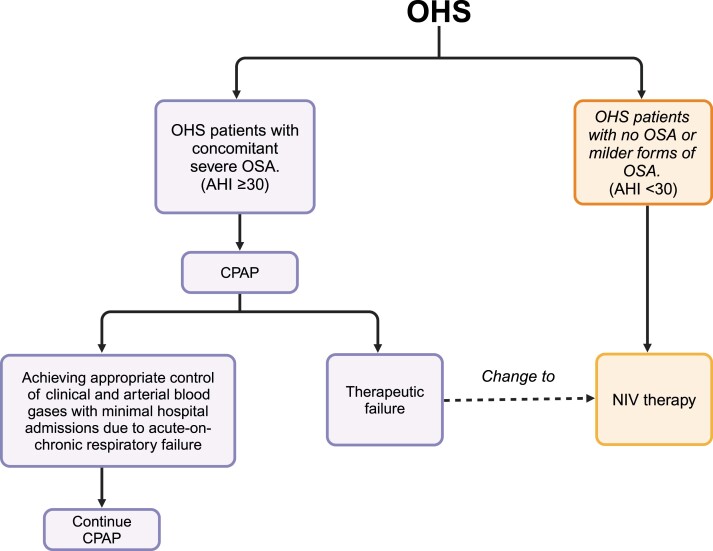
General management of OHS. Continuous positive airway pressure (CPAP) is the first-line treatment for OHS patients with severe obstructive sleep apnea (OSA). For OHS patients with no OSA or milder forms of OSA, noninvasive ventilation (NIV) should be considered the primary therapy. If patients initially treated with CPAP do not respond positively to therapy despite objectively documented high levels of adherence to CPAP, they should be switched to NIV therapy. AHI, apnea–hypopnea index.

Currently, no established protocol for managing OHS exists, and treatment is typically centered on correcting SDB, reducing weight, and managing comorbidities (Figure 3). In clinical practice, the management of patients may vary according to the acuity of the condition (Figure 4). Specifically, the approach to managing a patient with stable OHS differs from that of a patient exacerbated in an emergency room; in this case, it is crucial to systematically rule out potential complications, including pulmonary thromboembolism, pneumonia, or acute respiratory acidosis [51]. These conditions should be treated immediately to prevent any further complications.

Treatment of OHS should consider comorbid conditions, and it is important to recommend goals to be achieved and addressed, one of which is cardiorespiratory rehabilitation as well as targeted weight loss. “The addition of the rehabilitation program produced significant improvements in both functional performance and exercise capacity as well as increases in muscle size and strength, again supporting the enhanced effect of rehabilitation in addition to NIV” [52].

### Correction of SDB

The SDB can be treated with the use of positive pressure devices. The efficacy of these devices was initially documented in 1982, and since then, clinical trials have validated their effectiveness. PAP therapy CPAP or various modes of NIV is the principal treatment modality in patients, with SDB NIV consists of the application of positive-pressure ventilation, usually with Bi-level pressure settings, and CPAP consists of a continuous pre-set pressure during the respiratory cycle to prevent obstructive apneas and hypopneas [12, 53].

CPAP is the primary mode of treatment for OHS cases with concomitant severe OSA. However, in cases where CPAP is ineffective, Bi-level PAP may be necessary, particularly at the onset of treatment due to hypercapnia during acute exacerbation or hospitalization with decompensation. It is important to emphasize that NIV should be considered as first-line therapy for patients with no OSA or milder forms of OSA. If patients initially treated with CPAP have no favorable response to therapy despite objectively documented high levels of adherence to CPAP, they should be changed to NIV therapy [12].

On the other hand, according to a study by Masa et al., patients with OHS adopting NIV or CPAP can significantly alleviate hypercapnia symptoms during wakefulness. Interestingly, studies have shown that both long-term NIV therapy and CPAP treatment are equally effective, regardless of the severity of baseline hypercapnia. As such, clinicians can confidently prescribe CPAP treatment to this patient group, regardless of their initial PaCO2 levels; however, they must be reassessed to ensure the hypercapnia is resolved [54].

A prospective study was conducted on patients with stable OHS and undergoing positive pressure treatment to evaluate the efficacy of CPAP and Bi-level PAP. The study demonstrated that CPAP effectively eliminated respiratory events in 57% of patients, while the remaining patients required Bi-level PAP due to persistent hypoxemia or a high residual AHI. Notably, patients who did not respond to CPAP had a significantly higher BMI of 61.6 ± 17 kg/m2 compared to those who responded 56.5 ± 1.2 kg/m2 [55].

In 2008, Piper et al. reported no significant differences in treating patients with either CPAP or Bi-level PAP if adherence was equal and improvements in excessive daytime sleepiness, hypoxemia, and hypercapnia were observed. Additionally, the study showed that up to 80% of patients responded adequately to CPAP, indicating that it is an effective treatment option for most patients with OHS [56].

Therefore, it is strongly suggested that CPAP be titrated at the outset of treatment, given the higher costs associated with Bi-level PAP and the evidence presented in the literature. However, the evaluation of each patient’s unique clinical scenario is imperative. The use of Bi-level PAP should be considered when a patient is unable to tolerate high pressures, when hypoxemia and/or hypoventilation persist despite eliminating obstructive events, and when the patient continues with hypercapnia following three months of CPAP treatment [56, 57].

In positive pressure therapy, the administration of oxygen becomes necessary when the patient, despite overcoming various respiratory events, maintains a saturation below 88% at sea level. Oxygen supplementation is required if T90 exceeds 10% at sea level. Recent studies have shown that sleep-related hypoxemia, which measures T90, is correlated with cardiovascular events. T90 is classified into four categories: light (T90 ≤ 5%), mild (T90 5%–10%), moderate (T90 10%–25%), and severe (T90 > 25%). This classification helped stratify the risk of hypertension in OSA patients. In the case of Mexico City, located at an altitude of 2400 meters above sea level, the threshold for oxygen supplementation is set at a maximum T90 of 30% [57].

Patients who adhere well to positive pressure therapy have improved their quality of life after six months of CPAP treatment [41].

Moreover, CPAP or Bi-level PAP treatment has been shown to be effective in improving arterial blood gases in patients experiencing respiratory disorders. The efficacy of other positive pressure treatments in such patients has yet to be well established. As stated earlier, in cases where CPAP is ineffective, Bi-level PAP may be necessary, particularly at the onset of treatment due to the presence of hypercapnia. Bi-level PAP may also be better tolerated by patients, particularly when the CPAP pressures required to eliminate respiratory events are unmanageably high [12]. However, in cases where the patient does not respond adequately to CPAP or Bi-level PAP, alternative therapies such as average volume-assured pressure support (AVAPS) can be considered [58]. AVAPS integrates the characteristics of both volume and pressure-controlled noninvasive ventilation. In Bi-level PAP mode, volume is the dependent variable, whereas in AVAPS mode, pressure is the dependent variable [59]. Bi-level PAP substantially improves clinical parameters such as oxygenation, sleep quality, and health-related quality of life in patients with OHS. The addition of AVAPS to BPV-S/T provides additional physiologic improvements and benefits on ventilation quality, thus resulting in a more efficient decrease of transcutaneous PCO2 compared to BPV-S/T therapy alone. This, however, did not provide further clinical benefits regarding sleep quality and health-related quality of life in the present group of highly selected patients [58].

### Treatment approaches based on OHS phenotype

The selection of an appropriate treatment strategy for patients with OHS can be a challenging task. Treatment choice largely depends on the phenotype, which encompasses various types of breathing abnormalities during sleep. When hypoventilation predominates over obstructive events during sleep, NIV modalities such as Bi-level PAP are considered an appropriate treatment strategy. Conversely, for patients with a higher frequency of obstructive events during sleep, CPAP is a recommended first-line treatment. Patients on CPAP should be closely monitored for 2–3 months, and in case of treatment failure (when CO_2_ levels do not normalize), NIV modalities such as Bi-level PAP should be considered [60].

### Other treatments

It is imperative to educate patients with OHS about the importance of weight control and encourage them to make necessary lifestyle modifications and implement rehabilitation strategies. Although the long-term efficacy of these measures remains yet to be established, it is crucial to keep the patients informed and motivated. Bariatric surgery can be considered a viable alternative for patients who meet the criteria. However, it is essential to exercise caution while recommending this option and consider the potential risks involved. Sugerman et al. studied 61 patients with OHS who underwent bariatric surgery. After one year, 31 patients improved PaO2 (from 53 to 73 mmHg) and PaCO2 (from 53 to 44 mmHg). However, at the 5-year follow-up, only 12 patients underwent arterial blood gas analysis, which showed a significant decline in their PaO2 levels (mean PaO2 = 68 mmHg) and an increase in PaCO2 levels (mean PaCO2 = 47 mmHg). Additionally, their BMI had increased from 38 to 40 kg/m2 since the first year after surgery. The success of bariatric surgery, including weight loss and physiological parameters, depends on patient-specific variables and surgical techniques. When considering weight loss surgery, it’s important to carefully weigh the potential risks of the procedure against the maximum level of anticipated weight loss. It is worth noting that even after weight reduction surgery, OSA may persist despite the resolution of OHS [48].

There is an increasing amount of research being conducted on the use of incretins for managing obesity. These studies aim to determine the effectiveness of incretin therapy in reducing cardiovascular risks and other complications associated with excess body fat. Despite recent studies showing significant weight loss, there are still many unanswered questions about the use of incretin therapy as a treatment for OHS. Current evidence suggests that discontinuing incretin treatment may result in weight regain and the eventual reversal of cardio-metabolic improvements [61].

The use of respiratory stimulants, such as medroxyprogesterone and acetazolamide, has generated considerable debate. Medroxyprogesterone acetate acts as a respiratory stimulant in the hypothalamus. Progestins have been found to improve hypercapnia and hypoxemia in patients with OHS, but not completely eliminate it. On the other hand, acetazolamide prevents the conversion of carbon dioxide to bicarbonate, which lowers pH in the brain and increases central ventilatory drive and minute ventilation. It has been shown to improve AHI, increase PaO2, and reduce PaCO2 in patients with OSA [62]. However, stimulants can increase negative intrathoracic pressure and promote upper airway collapse. Therefore, it is prudent to approach the use of these stimulants with due diligence and weigh the potential benefits against the possible risks involved. It is important to note that the use of medroxyprogesterone can increase the risk of venous thromboembolism. Due to this, it is not recommended to administer the medication to patients with OHS who have limited mobility. Similarly, when prescribing Acetazolamide to patients with ventilatory limitations, caution should be exercised as the drug can exacerbate acidosis and worsen dyspnea [63]. It is important to avoid using supplemental oxygen in isolation and instead adopt a comprehensive and integrated approach for treating patients with OHS [60].

### Treatment of the exacerbated patient

The administration of NIV represents a viable approach to reducing the need for emergency hospitalizations in patients exhibiting exacerbation [26]. In cases where the patient is already hospitalized, prompt identification of the syndrome is essential, and treatment with NIV should be initiated without delay. Empirical recommendations favor using the Bi-level PAP based on arterial blood gases. Polysomnography is performed following hospital discharge to titrate CPAP or Bi-level PAP. Whilst NIV is important in managing exacerbation; it is imperative to establish the causes of exacerbation (such as acute coronary syndrome, pulmonary embolism, and pneumonia) and identify any possible complications in all hospitalized patients [64]. During an acute event, NIV should be adjusted to manage upper airway obstruction. Expiratory PAP should be titrated for this purpose. Meanwhile, inspiratory PAP should be titrated to maintain adequate tidal volumes and lower PaCO2 [12]. Phlebotomy should only be considered in patients exhibiting hematocrit levels exceeding 65% and symptomatic hyperviscosity [65].

## Preoperative Assessment

Proper preoperative assessment is essential for minimizing post-surgical complications in all patients. However, patients with OHS pose a particular challenge to anesthesiologists and internists due to the fact that OHS and comorbidity are often undiagnosed or untreated in patients who undergo surgery; this is associated with a higher risk of morbidity. In a cohort study by Roop Kaw et al., involving 1800 patients with a BMI exceeding 30 kg/m² who underwent polysomnography, they found 194 patients with OHS who underwent elective non-cardiac surgery. The study demonstrated that patients with OHS are at an increased risk of postoperative complications, including respiratory failure in 21% of patients, heart failure in 8%, prolonged intubation in 13%, reintubation in 6%, tracheostomy in 2%, transfer to the ICU in 21%, and mortality rates of 1% within 30 days after surgery and 5% within a year [66]. These findings highlight the importance of thorough preoperative evaluation in patients with OHS who are scheduled for surgery to prevent any complications during or after surgery. As the initial step in the preoperative assessment, it is imperative to perform a comprehensive history and clinical examination to identify OHS along with its comorbidities. To ascertain patients at an elevated risk of developing complications, it is recommended to request measurements of serum SpO2 and bicarbonate levels along with the AHI [67].

A systematic review has recommended using the STOP-Bang questionnaire for surgical patients. This questionnaire is a combination of two question sets: the STOP questionnaire, which evaluates snoring, tiredness, observed apneas, and elevated blood pressure, and the Bang questionnaire, which assesses BMI ≥ 35, age over 50, neck circumference over 40 cm, and male gender [68]. A positive screening result, characterized by three or more affirmative responses, SpO2 levels below 90%, and elevated bicarbonate levels, indicates a heightened likelihood of postoperative adverse events [67].

OHS patients are at an increased risk of developing congestive heart failure and pulmonary hypertension. Therefore, during physical examinations, clinicians should be vigilant for suggestive signs. A 12-lead electrocardiogram should be obtained to search for right ventricular hypertrophy, while signs of cardiomegaly or pulmonary hypertension should be ruled out during chest x-rays. In cases where obese patients are unable to perform physical activity, cardiopulmonary capacity levels must be evaluated through pharmacological stress testing and transthoracic echocardiogram [62].

In cases where patients present symptoms alongside chronic obstructive pulmonary disease, it is of utmost importance to conduct spirometry tests as a means of diagnosing the conditions and reducing the likelihood of postoperative complications. Furthermore, obese patients exhibiting hypoxemia during wakefulness or demonstrating elevated levels of serum bicarbonate should undergo arterial blood gas measurements, which can confirm the presence and ascertain the severity of daytime hypercapnia [62].

Perioperative measures for individuals with OHS include several critical steps, namely, the early resumption of PAP therapy, prompt emergence from anesthesia, meticulous airway management, and vigilant monitoring to identify any respiratory complications that may arise. These steps are essential to ensure the safety and well-being of patients with OHS undergoing surgery [69].

Proper monitoring of patients in the post-anesthetic care unit is crucial to identify any recurring respiratory events such as apnea lasting more than 10 seconds, bradypnea with less than eight breaths per minute, mismatch between pain and sedation, CO2 retention or oxygen desaturation below 90%. This monitoring also helps to identify patients who are at a high risk of postoperative respiratory complications and need increased monitoring [62, 67, 70].

Venous thromboembolism (VTE) is a significant postoperative complication that requires appropriate prophylaxis to minimize the risk of morbidity and mortality. To accomplish this goal, healthcare providers recommend a combination of mechanical and pharmacologic interventions, including the use of thromboembolic stockings, sequential alternating compressive devices, low-dose subcutaneous unfractionated heparin or low molecular weight heparin (LMWH), and early postoperative mobilization. The optimal dosage and duration of treatment should be tailored based on the patient’s individual risk factors. In obese patients, LMWH may be administered once or twice daily, with fixed dosages being considered. However, there is no scientific evidence to support the need for twice-daily dosing for VTE prophylaxis. In addition, an increased dosage of LMWH, adjusted to BMI, may be appropriate in obese patients with a high risk of VTE, such as men, older patients, those with a high BMI, OSA, OHS, or a previous history of VTE, as long as there is no increased risk of bleeding [71].

## Conclusion

This review provides a thorough examination of OHS, a medical condition characterized by the coexistence of obesity and chronic hypoventilation. OHS is associated with significant morbidity, mortality, and healthcare expenses. We conclude that early identification and targeted management strategies such as weight loss, PAP therapy, and oxygen therapy are crucial for improving the prognosis of this illness. It is essential to combat the global obesity epidemic to reduce the increasing impact of OHS on public health. Further research is needed to develop more effective treatments and refine diagnostic criteria for this disease.

## Areas for Future Research

Performing a thorough preoperative evaluation is crucial in minimizing postoperative complications, especially in patients with OHS. To ensure a smooth surgery and recovery, conducting a multivariate analysis to establish a pretest scale of surgical risk for these patients could prove advantageous. Additionally, this could serve as a research opportunity and contribute to reducing the risk of morbidity resulting from delayed diagnosis.

## Data Availability

The data used to support the findings of this study are available from the corresponding author upon request.

## Notes

This work was performed in the Pneumology Service from the Institute of Security and Social Services for State Workers, Aguascalientes, México.
